# Beneficial effect of artificial intelligence care call on memory and depression in community dwelling individuals with dementia

**DOI:** 10.1038/s41598-025-12895-7

**Published:** 2025-07-25

**Authors:** Sungwoo Kang, Yunjin Lee, Nayoung Kim, Sang-Houn Ok, Pilsu Kim, Ok-Hyun Park, Da-Mi Kim, Yongwoo Jang, Hee-Jin Kim

**Affiliations:** 1 Department of Neurology, Hanyang University College of Medicine, 222 Wangsimni-ro, Seongdong-gu, Seoul, 04763 Republic of Korea; 2 Department of Medical and Digital Engineering, Hanyang University, 222 Wangsimni-ro, Seongdong-gu, Seoul, 04763 Republic of Korea; 3 NAVER Cloud, 6 Buljeong-ro, Bundang-gu, Seongnam, 13561 Gyeonggi Republic of Korea; 4 Seongdong-gu Dementia Safety Center, 3, Wangsimni-ro 5-gil, Seongdong-gu, Seoul, 04709 Republic of Korea; 5 Department of Pharmacology, Hanyang University College of Medicine, Seoul, 04763 Republic of Korea

**Keywords:** Artificial intelligence, Large language model, Intervention, Cognition, Depression, Geriatrics, Therapeutics

## Abstract

Traditional dementia-prevention programs have limited accessibility and lack continuous monitoring, making sustained cognitive and emotional support challenging. This study evaluated the effects of artificial intelligence (AI) care calls on the cognitive function and depression of community-dwelling individuals. Eighty participants with dementia received AI care calls twice weekly from April 23 to November 28, 2024, with 63 calls per participant. The AI care call provided emotional support and cognitive engagement. Cognitive function (attention, memory, and language) and geriatric depression scale (GDS) scores were assessed before and after the intervention. The Wilcoxon signed-rank test was used to compare the pre- and post-intervention scores. General linear models were used to examine the effects of age, sex, and education on changes in clinical measures while controlling for baseline clinical scores. After the AI care call intervention, GDS scores decreased significantly, while memory scores increased significantly. An interaction effect between age and sex was observed in attention and memory score changes, with females showing greater memory improvement and maintenance of attention scores, whereas males experienced a decline in attention scores with no significant change in memory. Additionally, an interaction effect between age and education was observed in the GDS and attention score changes, suggesting that higher education was associated with a protective effect against the worsening of these symptoms following the AI care call intervention. AI care calls may help reduce depression and improve memory function in community-dwelling individuals. The differential effects of sex and education suggest that these factors affect the effectiveness of interventions.

**Supplementary Information:**

The online version contains supplementary material available at 10.1038/s41598-025-12895-7.

## Introduction

The number of people with dementia is projected to rise from 57.4 million cases globally in 2019 to 152.8 million cases by 2050^1^. This increase is particularly pronounced in low- and middle-income countries owing to a faster increase in life expectancy compared to high-income countries^2^. Currently, drug therapy remains the primary approach for dementia treatment, but it has several limitations. Low medication adherence is a major challenge, as cognitive impairment hinders the ability of the patients to recognize and follow prescriptions^3^. Additionally, financial constraints make it difficult for economically challenged individuals. Although disease-modifying therapies, such as lecanemab^4^, have emerged, their clinical application remains limited owing to strict inclusion criteria and high costs, making them impractical for widespread clinical use^5^. Given these barriers, nonpharmacological interventions are gaining attention^5^, with artificial intelligence (AI)-driven solutions emerging as promising approaches for dementia care^6^.

Depression is also an important target in dementia care. It is associated with two-fold risk of dementia, and effective treatment of midlife depression may help reduce the future risk of developing dementia^5^. In individuals with dementia, depression not only contributes additional distress and reduced quality of life, but also exacerbates cognitive and functional impairment, increases mortality, and contributes to caregiver burden and institutionalization^7^.

AI chatbots provide structured, automated cognitive behavior therapy effectively to reduce symptoms of depression and anxiety in college students^8–10^. Similarly, recent meta-analyses have suggested that AI socially assistive robots have a beneficial effect on cognitive function in cognitively impaired individuals^11^. Although these interventions rely on rule-based interactions, recent advances have enabled large language model (LLM)-based AI chatbots to generate more dynamic and context-aware conversations. However, no studies have evaluated the effect of LLM-based AI chatbots on cognitive function or depression in older adults.

CLOVA CareCall is an LLM-based AI chatbot developed by Naver^12^, designed to provide personalized conversations, emotional support, and health monitoring for individuals, particularly the older individuals. It incorporates voice-based phone call interactions, simplified conversation flow, personalized memory features, and daily health check-ins tailored to the needs and limitations commonly experienced by this population. It utilizes *HyperCLOVA*^13^, an advanced Korean-language AI model, to engage users in natural, context-aware dialogue and assist in tracking their well-being.

In this study, we investigated changes in cognitive function and depression scores before and after the CLOVA CareCall intervention, which was conducted twice per week for 63 sessions in community-dwelling individuals with dementia. Additionally, we examined the interplay between age, sex, and education on clinical changes to understand how these factors influenced the effectiveness of the CLOVA CareCall intervention.

## Results

### Baseline demographic and clinical characteristics of study participants

Eighty participants (17 males and 63 females) were enrolled in this study (Table 1). The mean age was 79.9 ± 7.1 years (mean ± standard deviation), and the mean years of education was 5.2 ± 5.0 years. Of the participants, 59 (73.8%) lived alone, whereas the remaining 21 (26.3%) lived with their partners. Among the 63 participants with available clinical dementia rating (CDR) data, 59 (93.7%) had a CDR score 1, while only tree participants scored 2 and one participant scored 3. The baseline mean geriatric depression scale (GDS) score was 8.2 ± 4.8. The mean baseline scores for attention, memory, and language were 3.0 ± 1.5, 3.5 ± 3.4, and 2.5 ± 1.1, respectively.

**Table 1:** Baseline demographic of study participants.

Number	80
Age	79.9 ± 7.1
Female, N (%)	63 (78.8%)
Education	5.2 ± 5.0
Housing type	
Single-person household, N (%)	59 (73.8%)
Couple household, N (%)	15 (18.8%)
Others, N (%)	6 (7.5%)
Clinical dementia rating (*N* = 63)*	
1	59 (93.7%)
2	3 (4.8%)
3	1 (1.6%)
GDS score (0–15)	8.2 ± 4.8
Attention score (0–5)	3.0 ± 1.5
Memory score (0–10)	3.5 ± 3.4
Language score (0–4)	2.5 ± 1.1

Data are expressed as means ± standard deviation and number (%). The “others” category included individuals living with both a spouse and other family members (e.g., children) or those living with family members (e.g., children) without a spouse. GDS, geriatric depression scale. *Clinical dementia rating data were available for 63 out of the 80 participants.

### Changes in depression scale and cognitive scores after CLOVA CareCall

Table 2 presents the comparisons of the clinical outcomes before and 31 weeks after the CLOVA CareCall. After undergoing CLOVA CareCall, the GDS score significantly decreased from a median of 8.5 (Interquartile rage, IQR: 3.9 ~ 13.1) to 6.0 (IQR: 2.0 ~ 10.0). Specifically, at baseline, 34 participants (42.5%) scored ≥ 10 on the GDS score, indicating major depressive symptoms, while 21 participants (26.3%) scored between 5 and 9, reflecting minor depressive symptoms. Of the 55 participants with major or minor depressive symptoms at baseline, 15 of 34 (44.1%) with major depressive symptoms improved to minor or non-depressed status, and 9 of 21 (42.9%) with minor depressive symptoms transitioned to non-depressed status following the intervention. This distributional change was statistically significant (Stuart-Maxwell test, *P* = 0.002; Supplementary Table S1). The memory score significantly increased from a median of 3.0 (IQR: −0.5 ~ 6.5) to 4.0 (IQR: 0.9 ~ 7.1). There were no significant changes in the attention or language scores after receiving an AI care call.

**Table 2:** Comparison of pre- and post-intervention scores.

Test	Pre	Post	*P* value
GDS score	8.5 (3.9, 13.1)	6.0 (2.0, 10.0)	**< 0.001**
Attention score	3.0 (2.0, 4.0)	3.0 (2.0, 4.0)	0.417
Memory score	3.0 (− 0.5, 6.5)	4.0 (0.9, 7.1)	**0.021**
Language score	3.0 (2.5, 3.5)	3.0 (2.5, 3.5)	0.541

Values are presented as median (interquartile range). The Wilcoxon signed-rank test was used to assess the statistical significance of the pre- and post-intervention differences. False discovery rate (FDR) correction was applied to account for multiple comparisons (*n* = 4). Statistically significant values after FDR correction are shown in bold font. GDS, geriatric depression scale.

### Associations of age, sex, and education with changes in depression scale and cognitive scores

Table 3 presents the effects of age, sex, and education on changes in GDS and cognitive scores. The interaction effect of sex × education was tested but was not significant for any outcome. Regarding GDS score change, among the two-way interactions of age × sex and age × education, only the age × education interaction was significant. As shown in Supplementary Figure S1, individuals with lower education exhibited less negative changes in GDS scores with advancing age, whereas those with higher education showed more negative changes in GDS scores with increasing age. For attention score change, both two-way interactions of Age × Sex and Age × Education were significant. To evaluate whether the effect of age on clinical outcomes was moderated by sex, comparisons were conducted between female and male participants. Supplementary Table S2 presents a comparison of the demographic and clinical characteristics of female and male participants. Female participants had fewer mean years of education and a higher proportion of single-person households than male participants. The mean age and clinical variables were comparable between the two groups. Supplementary Table S3 presents the comparisons of clinical outcomes before and after the CLOVA CareCall in the female and male groups. In the female group, after undergoing CLOVA CareCall, the GDS score significantly decreased from a median of 9.0 (IQR: 4.3 ~ 13.8) to 6.0 (IQR: 2.0 ~ 10.0), whereas the memory score significantly increased from a median of 3.0 (IQR: −0.5 ~ 6.5) to 5.0 (IQR: 2.0 ~ 8.0). There were no significant changes in the attention or language scores in the female group after receiving the CLOVA CareCall. In the male group, although the median GDS score remained at 8.0 (IQR: 3.5 ~ 12.5 pre-intervention vs. 4.0 ~ 12.0 post intervention), the mean GDS score significantly decreased (Supplementary Figure S2), indicating an overall decline in GDS score following the intervention. The attention score significantly decreased from a median of 3.0 (IQR: 2.0 ~ 4.0) to 2.0 (IQR: 1.5 ~ 2.5). There were no significant changes in the memory or language scores in the male group after receiving the CLOVA CareCall. Regarding the interaction effect of Age × Education on attention score change, Supplementary Figure S1 illustrates that individuals with lower education exhibited a decline in attention score change with advancing age, whereas those with higher education showed an increase in attention score change with increasing age. Regarding memory score changes, only the two-way Age × Sex interaction was significant. As mentioned in Supplementary Table S3, memory scores increased in the female group, whereas in the male group, memory scores remained unchanged after receiving the CLOVA CareCall. There were no interaction effects of Age × Sex or Age × Education on language score changes.

**Table 3:** Associations of age, sex, and education with changes in clinical measures.

	Age		Sex		Education		Age × Sex		Age × Education	
	B	P value	B	P value	B	P value	B	P value	B	P value
GDS score change	0.35	0.076	0.02	0.887	3.94	**0.012**	–	N.S	− 3.78	**0.014**
Attention score change	− 0.32	**0.013**	− 2.75	**0.008**	0.08	0.480	2.44	**0.016**	–	–
Attention score change	− 0.56	**0.003**	− 0.31	**0.004**	− 3.84	**0.009**	–	–	3.89	**0.007**
Memory score change	− 0.24	0.099	− 2.33	**0.039**	0.002	0.985	2.20	**0.049**	–	N.S
Language score change	− 0.04	0.738	0.08	0.453	− 0.06	0.605	–	N.S	–	N.S

The results were based on general linear models for clinical measures, using age, sex, and education as the main predictors while controlling for baseline clinical scores. Only one interaction term (Age × Sex, Age × Education, or Sex × Education) was included at a time in each model. If the interaction term was significant, it was retained in the model. Otherwise, only the main effects of age, sex, and education were considered. The Sex × Education interaction was tested but was not significant for any outcome and is therefore not reported in the table. GDS, geriatric depression scale; N.S, not significant.

## Discussion

In this study, we investigated the differences in GDS and cognitive scores before and after 31 weeks of CLOVA CareCall intervention in 80 community-dwelling older adults with dementia. Our major findings are as follows. First, the GDS scores significantly decreased, and the memory scores significantly increased after the CLOVA CareCall intervention. Second, an interaction effect between age and sex was observed in the attention and memory score changes, with females showing a protective effect on attention and greater increase in memory scores following the CLOVA CareCall intervention. Additionally, an interaction effect between age and education was observed in both the GDS and attention score changes, suggesting that higher education had a protective effect against these changes following the CLOVA CareCall intervention. Thus, when combined together, the CLOVA CareCall may be an effective nonpharmacological approach for improving memory function and reducing depressive symptoms in older adults with dementia. Moreover, the differential effects observed based on sex and education highlighted the potential role of individual factors in modulating intervention effectiveness, emphasizing the need for personalized approaches to AI-driven dementia care.

Our first major finding was that after 31 weeks of the CLOVA CareCall intervention, the GDS scores decreased, and memory scores increased. Although previous studies have shown the potential of chatbot-based and AI robot interventions to reduce depression, stress, and neuropsychiatric symptoms in older adults^14,15^, these were primarily rule-based systems. Similarly, chatbot-based cognitive training has demonstrated improved memory function and reduced depression and anxiety in older adults, with higher engagement and adherence leading to better outcomes^16^. However, these interventions often required active user participation through structured tasks or digital interactions. Our study is the first to demonstrate that an LLM-based CLOVA CareCall intervention can effectively improve depression and memory function through simple phone calls alone, highlighting its potential as a highly accessible and low-barrier solution for older adults with behavioral or cognitive impairment.

Interestingly, the cognitive benefit of the CLOVA CareCall intervention was limited to memory, with no improvements observed in attention or language domains. There are three possible explanations for this selective effect. First, memory impairment was more pronounced than in other domains (Table 1). Memory scores were lower (Mean = 3.5, standard deviation, SD = 3.4 on a 0–10 scale) compared to attention (Mean = 3.0, SD = 1.5 on a 0–5 scale) and language (Mean = 2.5, SD = 1.1 on a 0–4 scale). This greater impairment may have allowed more room for measurable improvement following the intervention. Second, the nature of the intervention itself may have selectively facilitated memory improvement through modulation of the default mode network (DMN). Previous studies have shown that cognitive training can enhance memory function and strengthen within-network connectivity, particularly in the DMN^17^, which plays a pivotal role in episodic memory processing^18^. Given that our intervention emphasized autobiographical recall and conversational engagement, it is plausible that it preferentially engaged the DMN, whereas other cognitive domain, such as attention and language, may require stimulation of different neural circuits not sufficiently targeted by the current intervention format. Third, the clinical response to the intervention may vary depending on the disease stage of the target population. A recent study of socially isolated older adults with normal cognition or mild cognitive impairment reported that internet-based conversational engagement improved attention and increased connectivity within the dorsal attention network (DAN), without significant changes in the DMN^19^. Given that reduced cerebellar connectivity of DMN and posterior connectivity within the frontal parietal network have been shown to differentiate Alzheimer’s disease (AD) from mild cognitive impairment (MCI), but not MCI from healthy control, it is possible that individuals with more advanced DMN disruption, such as those with dementia, may be more responsive to AI-based conversational interventions. Future studies are warranted to investigate how the nature of the intervention and the disease stage of the target population interact to shape cognitive benefits, as different types of interventions are known to differentially engage distinct brain networks^17^.

Our second major finding was that females showed greater cognitive benefits, including a protective effect on attention and enhanced memory improvement, whereas higher education was associated with a protective effect on changes in the GDS and attention scores after the CLOVA CareCall intervention. These findings are consistent with previous studies suggesting that non-pharmacological interventions provide greater cognitive benefits to women than to men^20–22^. Several hypotheses may explain this difference. One possibility is that older women, particularly those from earlier cohorts, had fewer educational opportunities^23^, which aligns with our results (Supplementary Table S2), leaving more room for improvement through interventions. Additionally, women generally exhibit stronger verbal and episodic memories^24^, which may enhance their response to cognitive training. Regarding the protective effect of higher education on changes in GDS and attention scores, it is notable that previous studies have shown inconsistent results regarding the effect of education on the efficacy of cognitive intervention in depression^25,26^ and cognition^26,27^. Unlike structured cognitive training, which requires active learning and cognitive effort, the CLOVA CareCall intervention demanded sustained conversational engagement, which may have amplified the protective effect of education. Future studies should optimize the CLOVA CareCall for individuals with lower education levels by integrating multimodal support and personalized engagement strategies to enhance usability and intervention effectiveness.

This study has some limitations. First, the lack of a control group made it difficult to determine whether the observed changes resulted solely from the CLOVA CareCall intervention or were influenced by other factors. Second, the follow-up period was limited to 31 weeks, leaving the long-term effects of the interventions unknown. Future studies should assess whether the cognitive and emotional benefits persist beyond the intervention period. Third, unmeasured confounding factors, such as apolipoprotein ε4 status, lifestyle changes, caregiver support, medication adherence, and underlying medical conditions, may have influenced the results. However, our subgroup analyses based on living type showed that participants living alone experienced significant improvements in GDS score (Supplementary Table S4), suggesting robust utility of the AI intervention even among socially isolated older adults. Fourth, the study lacked comprehensive neuropsychological assessments and specific dementia diagnoses, limiting the ability to evaluate how the CLOVA CareCall affects different cognitive domains and dementia subtypes, such as AD or dementia with Lewy bodies. Future studies should incorporate detailed neuropsychological tests and biomarker-confirmed diagnoses to better understand the variability in responses to AI-driven dementia care.

## Conclusions

The LLM-based CLOVA CareCall intervention increased memory score and reduced depressive symptoms in community-dwelling older adults with dementia, suggesting its potential as an effective nonpharmacological approach. The differential effects of sex and education highlight the importance of personalized AI-driven dementia care.

## Methods

### Study participants

Eighty participants were recruited from the Seongdong-gu Dementia Safety Center in Seoul, South Korea, between April 2024 and November 2024. The inclusion criteria required participants to be home-dwelling individuals with dementia, defined as having a diagnosis of dementia through either clinical assessment at the Seongdong-gu Dementia Safety Center or external documentation. Of the total participants, 63 participants were clinically assessed at the center, where dementia severity was evaluated using the CDR^28^, and individuals scoring ≥ 1 were classified as having dementia. All CDR assessments were conducted by board-certified neurologists from the department of neurology at Hanyang University Seoul Hospital. The remaining 17 participants were registered based on external diagnoses, typically supported by a prescription containing a dementia diagnosis code, or less commonly, a formal medical certificate. Participants were prioritized in the following order: living alone, older couples, and others. The “others” category included individuals living with both a spouse and other family members (e.g., children) or those living with family members (e.g., children) without a spouse. The participants were also required to have no hearing impairments. All participants underwent baseline assessments before receiving the CLOVA CareCall, which was conducted twice weekly for 63 sessions, followed by follow-up assessments at the end of the intervention. Cognitive function was assessed in terms of attention, memory, and language function using the cognitive impairment screening test (CIST), while depression severity was measured using the GDS.

### Standard protocol approval, registration, and patient consent

This study was approved by the Institutional Review Board of the Hanyang University Seoul Hospital (IRB No: 2025-05-009-002). Written informed consent was obtained from all participants or their primary caregivers before the protocol-specific procedures were performed. Specifically, in cases where participants lived alone but had an identifiable primary caregiver (e.g., adult children or legal guardians), the caregiver was contacted either in person or by phone to explain the study and obtain consent. For participants without an available caregiver, trained staff at the Seongdong-gu Dementia Safety Center obtained consent directly from the individual after confirming their capacity to understand the study and provide voluntary agreement. This assessment included brief, structured communication to ensure that the individual could comprehend the purpose of the study and express a voluntary decision. Only individuals who demonstrated adequate understanding of the study and voluntarily agreed to participate were included in this study. All procedures were performed in accordance with the ethical standards of the 1964 Declaration of Helsinki and its amendments.

### CLOVA carecall

The first version of CLOVA CareCall was developed during the corona virus disease (COVID)-19 pandemic in March 2020. At that time, it functioned primarily as a chatbot-based AI call system^29^ for monitoring COVID-19 symptoms, specifically fever. Since the system operated via phone calls, it was found to be particularly suitable for elderly users, proving effective in COVID-19 monitoring and demonstrating its potential as a future elderly care system.

Building on this foundation, in November 2021, as shown in Supplementary Figure S3, the system was expanded using Naver’s LLM, *HyperCLOVA*. This enhancement allowed CLOVA CareCall to initiate phone conversations to check the well-being of elderly individuals living alone, demonstrating its effectiveness in public healthcare settings^30^. In August 2022, a memory function was added^31^, enabling the system to recall previous conversations. As of March 2025, the service has been deployed across 140 institutions nationwide, assisting approximately 30,000 users.

CLOVA CareCall operates as a system where a public institution’s staff member registers a recipient via a web-based platform, prompting AI to make periodic phone calls. The AI engages in conversations with the recipient and analyzes the responses, generating reports that are delivered to the institution’s staff. The primary goal is to assess the safety and health of elderly individuals living alone by inquiring about key aspects such as meals, sleep, health, exercise, and outdoor activities. Responses to each question are analyzed and categorized as either “Yes” or “No.” Additionally, the system enables personalized interactions by incorporating contextual continuity, as it references information from previous conversations. Although there is no fixed time limit, each conversation includes a limited number of turns, with average calls lasting about 1 min and 30 s, sufficient to assess the condition of elderly individuals living alone. If a user misses a call, CareCall automatically retries up to three times within a designated time window; if all attempts fail, the missed call is marked as “not received” and reported to the relevant public institution for follow-up. To accommodate individuals with cognitive impairment, the system uses slow, clear speech and simple, intuitive language, and is currently limited to users who can participate in basic verbal interaction.

CLOVA CareCall was designed with privacy and security in mind from the initial system architecture stage, and the following measures have been implemented accordingly: (1) Memory Data Collection and Memory Function: The memory function of CLOVA CareCall is designed to support personalized conversations. The data retained in memory is strictly limited to categories related to daily life and health. It does not include personally identifiable information such as names, addresses, or medical diagnoses, and therefore cannot be used to directly identify individuals; (2) Data Storage and Access Control: All data is securely encrypted both at rest and in transit, in accordance with the Personal Information Protection Act of the Republic of Korea. Access to data is strictly restricted to authorized personnel, and all access is logged and subject to audit. Furthermore, data is permanently deleted once the retention period expires, the service is terminated, or the user withdraws consent; (3) Consent and Information Disclosure: Before using the service, users or their legal guardians are clearly informed about the purpose, scope, and retention period of data collection. Explicit consent is obtained, and users are allowed to withdraw consent at any time; and (4) Measures Against Misuse and Data Breaches: Since the stored data does not contain information that can identify individuals on its own, re-identification is not possible without access to separately secured information. Additionally, a dedicated privacy and security team conducts regular system audits and security inspections to ensure data protection. To illustrate the nature of the intervention, anonymized example transcripts of CLOVA CareCall conversations have been included as supplementary material (Supplementary Text S1).

### Depression and cognition assessments

Depression severity was quantified using the Korea version of the15-time GDS (total range: 0–15), which has demonstrated good reliability and validity in elderly Korean population^32^. Higher scores indicate greater severity of depression. Based on a recent Korean study^33^, cutoff scores of 5 and 10 were suggested for identifying minor depressive symptoms and major depressive symptoms, respectively.

Cognitive function was evaluated using the CIST^34^, a dementia-screening tool specifically developed for the Korean population. Briefly, the CIST is a short, structured interview assessing six cognitive domains, including orientation, attention, visuospatial function, executive function, memory, and language, with a total score ranging from 0 to 30, where higher scores indicate better cognitive performance. In this study, only the attention, memory, and language function subdomains were assessed. These domains were selected based on both practical and theoretical considerations: given the constraints of a community-based setting, including limited time, personnel, and financial resources, it was not feasible to administer the full battery. Moreover, memory, attention, and language are the domains most likely to exhibit near transfer effects^35^ in response to a telephone-based conversational intervention. Attention was assessed using five items related to time and place, with a total possible score of 0–5. Memory was evaluated based on a combined score of delayed recall (five items) and recognition (five items), with a total score ranging from 0 to 10. Language function was measured using four items assessing naming and comprehension, with the total score ranging from 0 to 4.

The assessments of CIST and GDS were administered during home visits conducted by trained case management nurses, who had received prior training in the standardized administration of these instruments.

### Statistical analysis

Statistical analyses of demographic and clinical data were conducted using R statistical software (version 4.2.1). Independent t-tests and χ^2^ tests were used to compare the demographics and clinical features between the female and male groups. The Wilcoxon signed-rank test was used to compare the clinical outcomes before and after the CLOVA CareCall. Changes in clinical outcomes were calculated by subtracting baseline scores from follow-up scores for each outcome measure. A positive change indicated an increase, whereas a negative value indicated a decrease over time. General linear models were applied while controlling for baseline clinical measures to investigate the effects of age, sex, and education on changes in clinical measures. Only one two-way interaction term (Age × Sex, Age × Education, or Sex × Education) was included at a time in each model. If the interaction term was significant, it was retained in the model; otherwise, only the main effects of age, sex, and education were analyzed.

## Supplementary Information

Below is the link to the electronic supplementary material.

Supplementary Material 1

## Data Availability

The datasets generated and/or analyzed during the current study are not publicly available due to sensitive personal information, but are available from the corresponding author on reasonable request and can be shared after anonymization.
