# Supplementary material for: Beneficial effect of artificial intelligence care call on memory and depression in community dwelling individuals with dementia
Source: Sci Rep. 2025 Jul 25;15:27116. doi: 10.1038/s41598-025-12895-7 (PMC12297396; doi:10.1038/s41598-025-12895-7)
Supplement: Supplementary file 1 — Supplementary Material 1 [file 41598_2025_12895_MOESM1_ESM.docx]

**Supplementary Figure S1.** Interaction effects of age and education on GDS and attention score changes

**
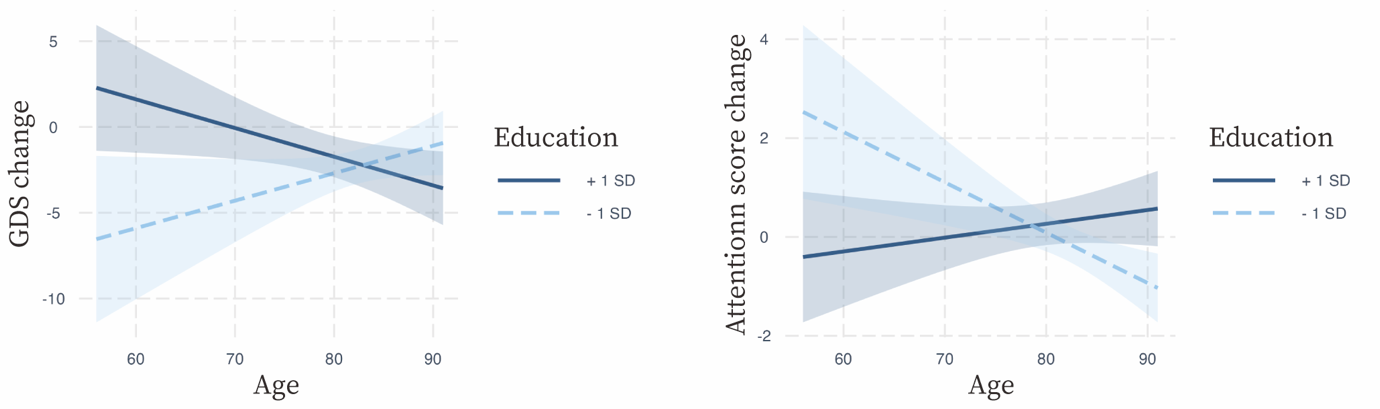
**

GDS, geriatric depression scale.**Supplementary Figure S2.** Comparison of pre- and post-Intervention GDS scores in male

**
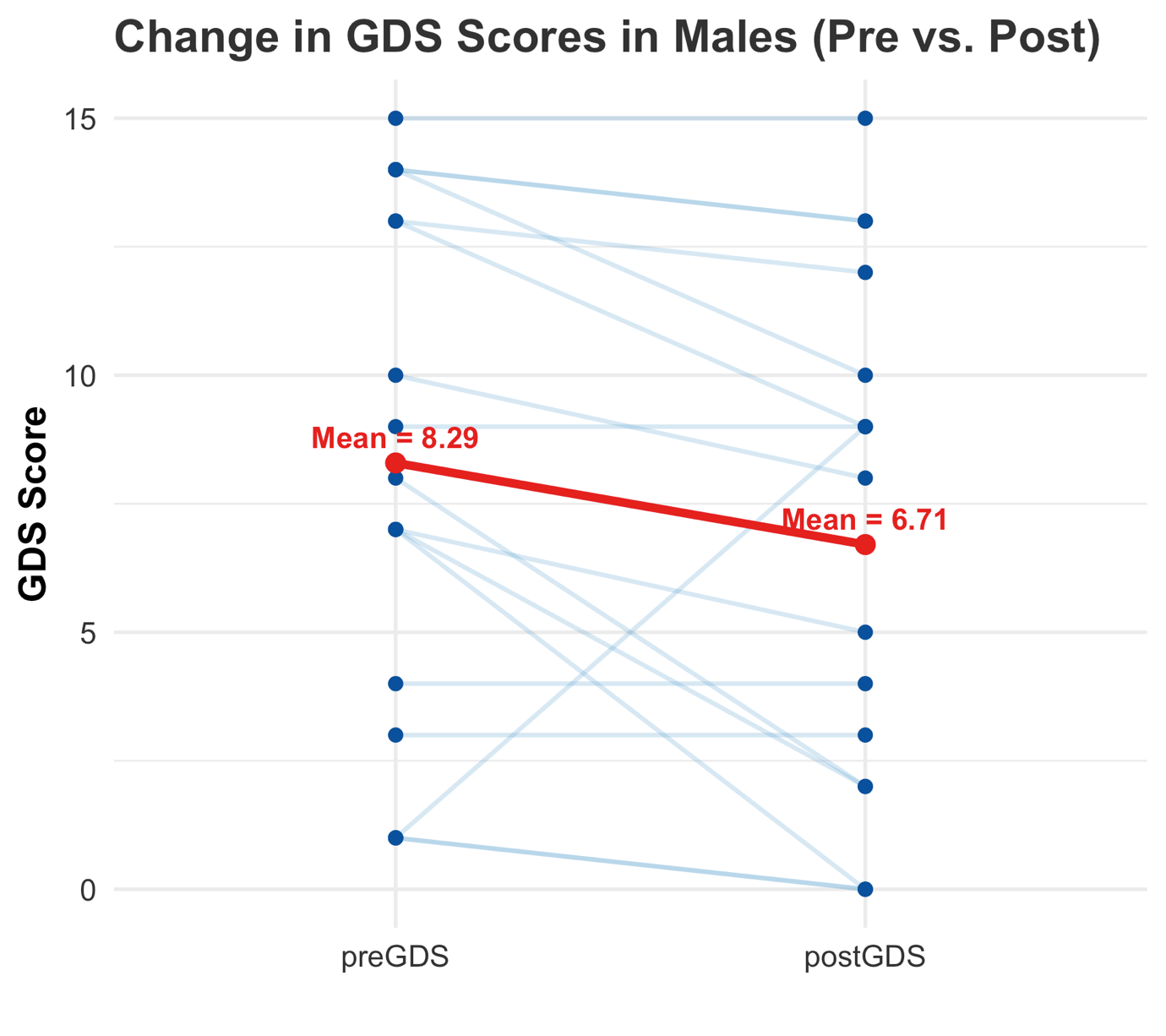
**

GDS, geriatric depression scale.

**Supplementary Figure S3.** Overview of CLOVA CareCall system and service

**
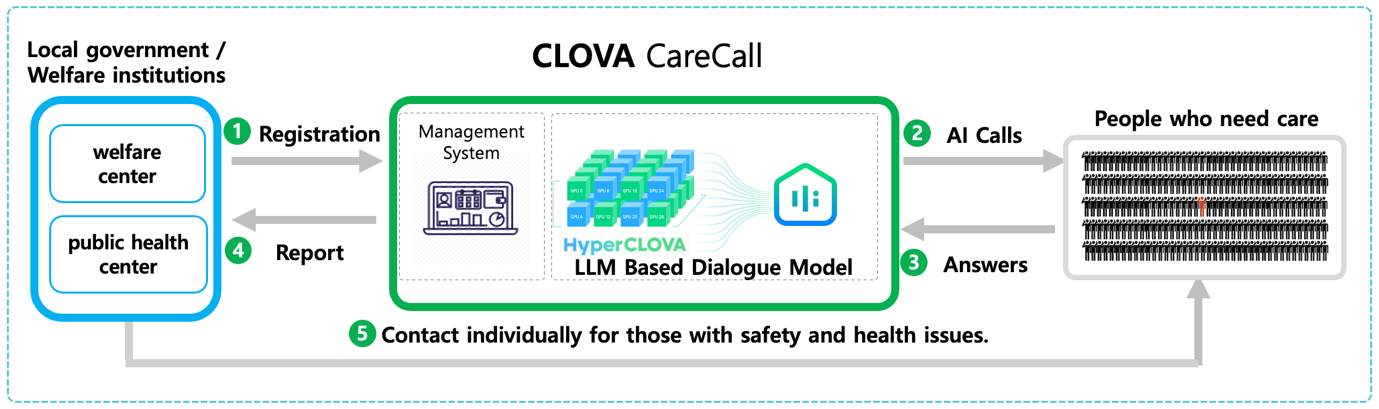
**Clova CareCall has built-in safeguards to identify and respond to high-risk situations, such as signs of abuse, neglect, suicidal ideation, or expressions of intent to harm others. Below is an outline of how such scenarios are handled: 1) **Detection via AI Conversation Monitoring**: The system uses natural language understanding (NLU) and keyword-based flagging mechanisms to detect language patterns or expressions that may indicate emotional distress, abuse, or self-harm. These include, but are not limited to, phrases related to violence, depression, loneliness, or fear; 2) **Triage and Alert Mechanism**: If such language is detected during the call, the conversation is automatically flagged as "high-risk" in the report generated for the responsible public institution. These reports are reviewed daily by case managers, allowing for rapid follow-up. In most municipalities, the standard protocol involves direct phone contact and, if necessary, a home visit by social workers or health staff; 3) **Limitations and Ethical Considerations**: While the AI can detect risk signals based on pre-trained patterns, it does not replace clinical judgment. Therefore, the flagged data serves as an early warning system rather than a diagnostic tool. The design prioritizes early detection while minimizing false positives to reduce unnecessary distress or intervention; and 4) **Human (Care Manager) Oversight and Response Protocol**: All flagged cases require human verification. Public officials or caregivers review the case and determine the appropriate action based on local protocol. This hybrid model ensures both scalability through automation and ethical safeguards through human oversight.

**Supplementary Table S1**. Pre- to post-intervention shifts in depression severity categories

| Post  Pre | Major-depression | Minor-depression | Non-depressed |
| --- | --- | --- | --- |
| Major-depression (N=34) | 19 | 9 | 6 |
| Minor-depression (N=21) | 3 | 9 | 9 |
| Non-depressed (M=25) | 0 | 3 | 22 |

Classification based on Geriatric Depression Scale scores: Major depressive symptoms ≥10, Minor depressive symptoms 5–9, Non-depressed ≤ 4. The table shows the number of participants who transitioned between categories from baseline to post-intervention. Statistical significance was assessed using the Stuart-Maxwell test for marginal homogeneity.

**Supplementary Table S2.** Baseline demographics of the study participants by sex

|  | Female (N=63) | Male (N=17) | P value |
| --- | --- | --- | --- |
| Age | 80.6 ± 6.5 | 77.1 ± 8.4 | 0.063 |
| Education | 4.3 ± 4.5 | 8.6 ± 5.3 | **0.001** |
| *Housing Type* |  |  |  |
| Single-person household, N (%) | 50 (79.4%) | 9 (52.9%) | **0.028** |
| Couple household, N (%) | 8 (12.7%) | 7 (41.2%) |  |
| Others, N (%) | 5 (7.9%) | 1 (5.9%) |  |
| GDS score | 8.1 ± 4.8 | 8.3 ± 5.0 | 0.891 |
| Attention score | 3.1 ± 1.5 | 2.9 ± 1.4 | 0.654 |
| Memory score | 3.7 ± 3.5 | 2.8 ± 3.1 | 0.356 |
| Language score | 2.4 ± 1.0 | 2.8 ± 1.2 | 0.177 |

Data are expressed as means ± standard deviation and number (%). The “others” category included individuals living with both a spouse and other family members (e.g., children) or those living with family members (e.g., children) without a spouse. GDS, geriatric depression scale.

**Supplementary Table S3**. Comparison of pre- and post-intervention scores by sex

|  | Pre | Post | P value |
| --- | --- | --- | --- |
| *Female (N=63)* |  |  |  |
| GDS score | 9.0 (4.3, 13.8) | 6.0 (2.0, 10.0) | **< 0.001** |
| Attention score | 3.0 (2.0, 4.0) | 3.0 (2.0, 4.0) | 0.951 |
| Memory score | 3.0 (−0.5, 6.5) | 5.0 (2.0, 8.0) | **0.035** |
| Language score | 3.0 (2.5, 3.5) | 3.0 (2.5, 3.5) | 0.583 |
| *Male (N=17)* |  |  |  |
| GDS score | 8.0 (3.5, 12.5) | 8.0 (4.0, 12.0) | **0.024** |
| Attention score | 3.0 (2.0,4.0) | 2.0 (1.5, 2.5) | **0.037** |
| Memory score | 1.0 (−2.0, 4.0) | 3.0 (0, 6.0) | 0.392 |
| Language score | 3.0 (2.0,4.0) | 3.0 (2.0, 4.0) | 0.821 |

Values are presented as median (interquartile range). The Wilcoxon signed-rank test was used to assess the statistical significance of the pre- and post-intervention differences. Statistically significant values are indicated in bold. GDS, geriatric depression scale.

**Supplementary Table S4**. Comparison of pre- and post-intervention scores by living type

|  | Pre | Post | P value |
| --- | --- | --- | --- |
| *Living alone (N=59)* |  |  |  |
| GDS score | 9.0 (4.5, 13.5) | 6.0 (1.8, 10.3) | **<0.001** |
| Attention score | 3.0 (2, 4) | 3.0 (2, 4) | 0.603 |
| Memory score | 4.0 (2.5, 3,5) | 3.0 (2.5, 3.5) | 0.058 |
| Language score | 9.0 (4.5, 13.5) | 11.0 (6.5, 15.5) | 0.351 |
| *Living with partners (N=21)* |  |  |  |
| GDS score | 7.0 (2.5, 11.5) | 5.0 (1.5, 8.5) | 0.154 |
| Attention score | 3.0 (1.5, 4.5) | 2.0 (1, 3) | 0.522 |
| Memory score | 0 (−2, 2) | 3.0 (1, 5) | 0.210 |
| Language score | 3.0 (2, 4) | 2.0 (1, 3) | 0.822 |

Values are presented as median (interquartile range). The Wilcoxon signed-rank test was used to assess the statistical significance of the pre- and post-intervention differences. Statistically significant values are indicated in bold. GDS, geriatric depression scale.

**Supplementary Text S1.** Example transcript of AI–participant conversation

This is a situation in which the senior and AI have a conversation, in the previous conversation AI recognizes that the old man was injured in the last conversation and asks questions about it in the next conversation.

**AI:** I remember you had a bloated stomach last time.

**User:** Yes! (feeling touched)

**AI:** How is your stomach today?

**User:** I feel a little better after taking some medicine. But I still feel a little bloated.

**AI:** Oh no… If you still feel bloated, I think you should visit the hospital.

**User:** Yes, I think that is a good idea.

**AI:** Yes, I hope you feel better soon

**User:** Thank you so much.
